# A Male Patient With Breast Hamartoma: An Uncommon Finding

**DOI:** 10.7759/cureus.9444

**Published:** 2020-07-28

**Authors:** Vincent T Phan, Nga T Nguyen, Jing He, Angelica S Robinson, Quan D Nguyen

**Affiliations:** 1 Radiology, University of Texas Medical Branch, Galveston, USA; 2 Pathology, University of Texas Medical Branch, Galveston, USA

**Keywords:** breast cancer, male breast tumor, cowden syndrome, male breast hamartoma, mammary hamartoma

## Abstract

Mammary hamartoma is a rare type of breast tumor that is composed of the same elements as normal mammary tissue. This condition is very rare in men. In current literature, there are fewer than five case reports on male breast hamartoma. This benign pathology is under-reported because of several reasons. Since breast tumors are still considered an exclusively female diagnosis and statistically proven to be gynecomastia when arising in men, they are often overlooked. In addition to the uncommon clinical presentation in men, insufficiency of definitive pathologic and radiologic characteristics can make an accurate diagnosis a challenging task. Mammary hamartoma is a benign condition with an excellent prognosis. The following case describes a rare instance of an enlarging mammary hamartoma in a male patient, highlighting the imaging features, pathohistological findings, and clinical management.

## Introduction

Breast cancer is the most concerning diagnosis to rule out in a patient with a breast tumor. Early detection of malignancy is key to initiate appropriate treatment that can prevent widespread metastases and poor outcomes. Over the last two decades, improved quality and increased use of diagnostic tools in male patients have elevated the rate of detection of breast conditions in men from 0.8% to 2.4%, which is significant as breast cancer in men accounts for 1% of all breast cancer cases [1]. Despite a recent rise in the number of male patients with breast complaints, breast tumors are much more common in women because of the increased breast tissue and differences in breast structures, hormones, and genetics. An international study comparing the incidence rates of male to female breast cancer between 1988 and 2002 revealed a female-to-male incidence rate ratio of 122 [2]. As a result, it is often believed that breast pathology is exclusively a woman’s health concern which leads to underdiagnosis within the male population.

Most breast masses in men are benign, with the most common diagnosis being gynecomastia. This benign pathology can be classified by sonography into nodular, dendritic, and diffuse glandular types and often appears as a smooth and well-circumscribed tumor [3]. Gynecomastia often presents as a unilateral or asymmetrical growth due to ductal and stromal proliferation and is typically located under the nipple. This benign pathology, often caused by a wide variety of medications or drugs, can affect a large proportion of the breast tissue. Gynecomastia may also be asymptomatic. Specifically, the prevalence of asymptomatic gynecomastia is 60% to 90% in neonates, 50% to 60% in adolescents, and up to 70% in men ages 50 to 69 years [4]. 

Theoretically, benign breast tumors can occur in both men and women; however, a breast hamartoma in a male patient continues to be a rare occurrence. Breast hamartomas account for 0.1% to 0.7% of all benign mammary masses, with the large majority of patients being middle-aged females [5]. Males with diagnosed breast hamartomas are mostly older than 35 years and rarely under 18 years with only a single reported case of a breast hamartoma with intrathoracic extension in a 13-year-old boy [6]. Oftentimes, breast hamartomas are incidentally seen on routine screening mammograms or on diagnostic mammograms of palpable breast masses in female patients. The incidence of this rare tumor in men is low with mammary hamartomas accounting for 0.12% to 0.24% of all male breast tumors [7]. A major contributing factor when considering the low frequency of breast pathologies in male patients is the likelihood of under-reporting. Due to the low incidence rate of breast tumors and differences in detection in men, physicians often dismiss the possibility of breast pathology in this patient population. Specifically, due to the health maintenance differences between men and women such as breast physical exams and routine screening mammograms, there may be fewer reported cases of hamartomas in men. This paper details the clinicopathologic findings and management of mammary hamartoma in a male patient. A literature review of imaging and histopathological characteristics of this rare pathology is also presented.

## Case presentation

**Figure 1:**
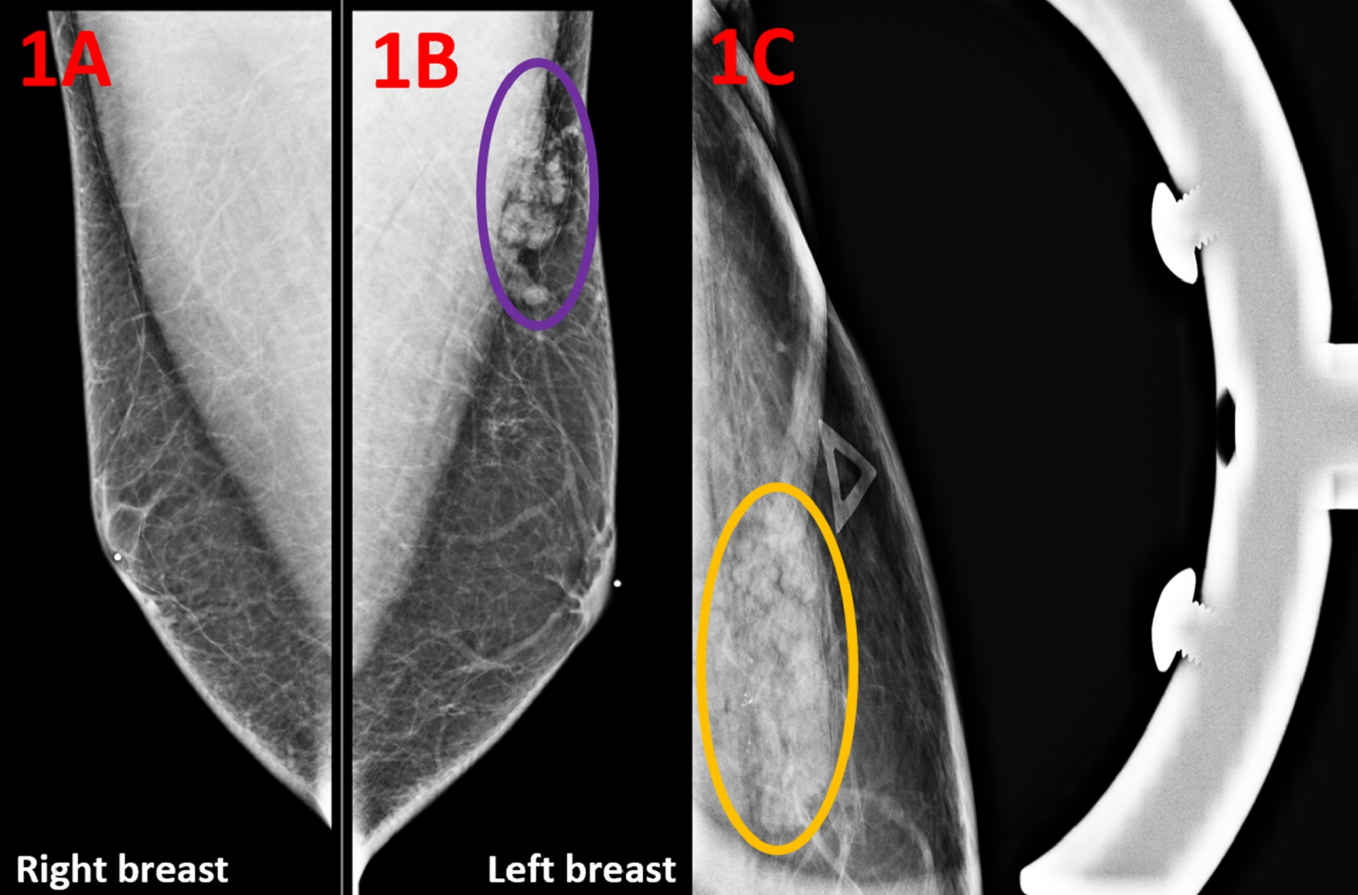
Diagnostic Mammogram of the Breasts in Mediolateral Oblique Views (A) Diagnostic mammography of the right breast shows no abnormality. (B) Diagnostic mammography of the left breast shows a high-density oval mass measuring 30 x 9 mm in the upper outer quadrant of the left breast (purple circle). (C) Compression diagnostic mammogram demonstrates a left breast upper outer quadrant mass with microlobulated margins and associated grouped coarse heterogeneous calcifications (yellow circle).

A 41-year-old man presented with a tender mass in the upper outer quadrant of the left breast. He reported having this mass since he was 26 years old and noted that it had been increasing in size over the past several months. His past medical history is significant for coronary atherosclerotic disease and depression. The patient denied any known family history of breast cancer. He previously smoked cigarettes, quantified to eight pack-years, but denied alcohol and illicit drug use. On physical examination of the right breast, no mass was palpated. On physical examination of the left breast, there was a solid mass at one o’clock at a distance of 10 cm from the left nipple. The normal right breast and the left breast mass can be visualized on diagnostic mammography (Figure 1A, 1B). Diagnostic mammogram spot compression was performed on the area of interest in the upper outer quadrant of the left breast, revealing a high-density oval mass with microlobulated margins and associated grouped coarse heterogeneous calcifications (Figures 1C).

**Figure 2:**
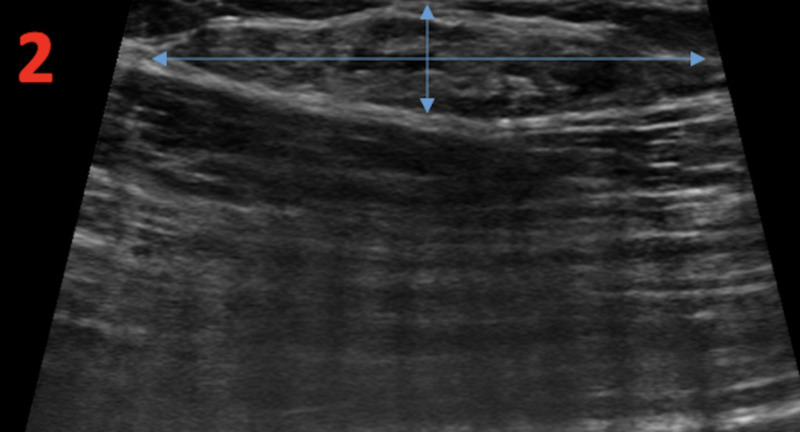
Left Breast Diagnostic Ultrasound Diagnostic ultrasound of the left breast demonstrates an isoechoic oval mass measuring 34 x 7 x 34 mm in the upper outer quadrant at a distance of 10 cm from the nipple (perpendicular blue arrows). The mass has circumscribed margins and internal vascularity.

Targeted ultrasound of the left breast showed a solid mass measuring 34 x 34 x 6 mm at the site of the mammographic and clinically palpable mass in the upper outer quadrant at one o’clock at a distance of 10 cm from the nipple (Figure 2). The characteristics of the imaging included an orientation parallel to the skin line and an oval shape. Increased vascularity was present.

**Figure 3:**
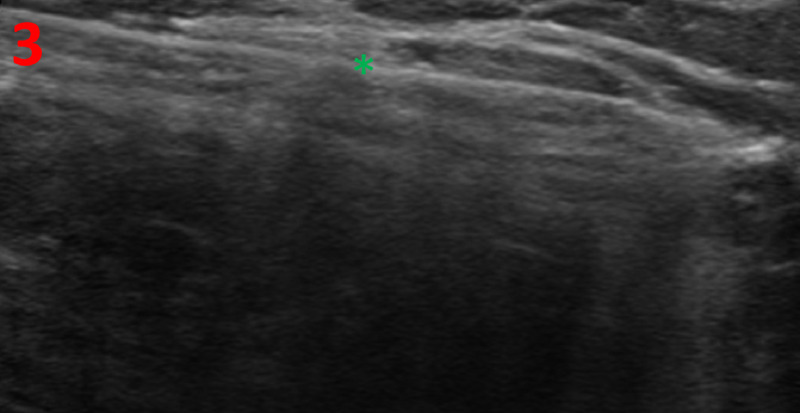
Left Breast Ultrasound-Guided Biopsy The ultrasound image shows a hyperechoic needle (green asterisk) through the mass in the upper outer quadrant at a distance of 10 cm from the nipple.

In consideration of the enlarging left breast mass and the abnormal imaging findings, an ultrasound-guided core needle biopsy of the left breast was performed for tissue diagnosis (Figure 3).

**Figure 4:**
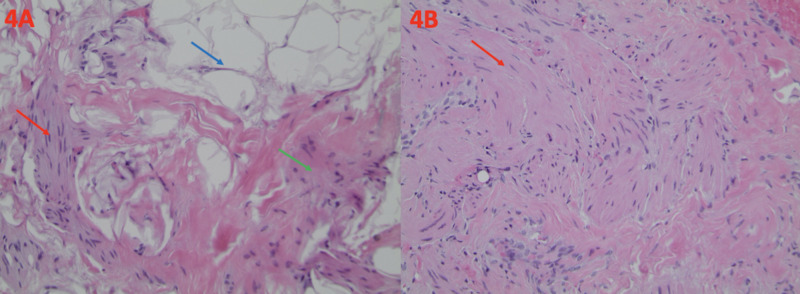
Histological Findings of Breast Hamartoma (A) The presence of smooth muscle bundles (red arrow), dense fibrous tissue (green arrow), and adipose tissue (blue arrow) is typical of breast hamartoma. This is demonstrated on a hematoxylin and eosin stain at a magnification of ×100. (B) Smooth muscle bundles are also present (red arrow). This is demonstrated on a hematoxylin and eosin stain at a magnification of ×200.

**Figure 5:**
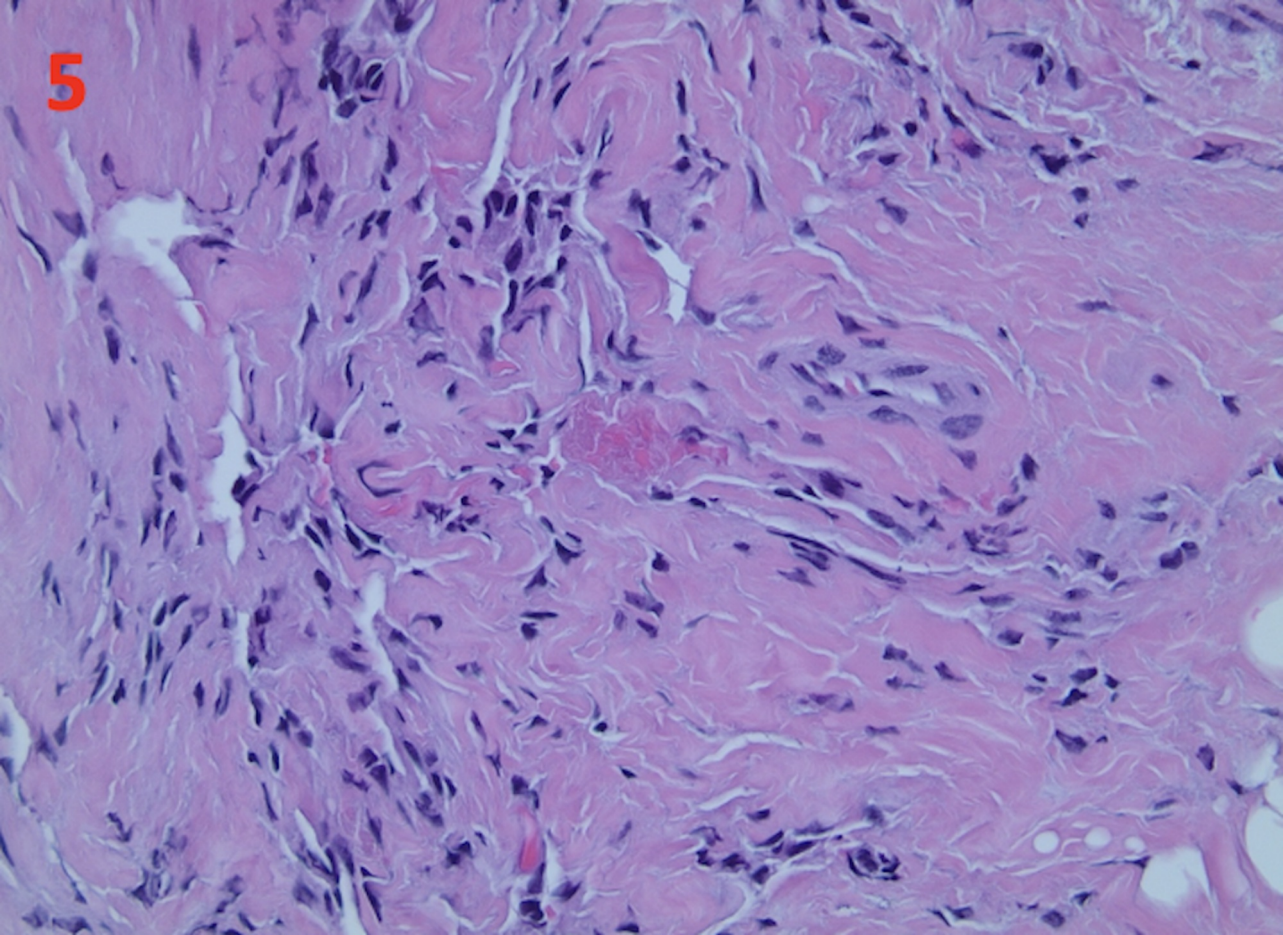
Pseudoangiomatous Stromal Hyperplasia in Breast Hamartoma The histology of the tumor biopsy shows pseudoangiomatous stromal hyperplasia. This is demonstrated at a magnification of ×400.

Histology of the biopsy revealed smooth muscle bundles, accompanied by fibrous stromal and adipose tissue, which is consistent with hamartoma (Figure 4). Pseudoangiomatous stromal hyperplasia was also present (Figure 5).

With the imaging and histology findings, a diagnosis of benign breast hamartoma was made without further clinical investigation. Reassurance and education were provided. The patient remained asymptomatic without any new breast masses at his follow-up health check a year later.

## Discussion

A hamartoma, also known as a fibroadenolipoma, lipofibroadenoma, or adenolipoma, is a benign and indigenous neoplastic growth [8]. Also described as “breast within a breast”, the tumor is a collection of fibrous, glandular, and adipose tissues with unique characteristics dependent on the surrounding indigenous tissue [9]. Clinically, hamartomas present as well-demarcated, encapsulated, and painless masses. Their sizes range from 1 to 8 cm in diameter. They are firm and rubbery, and typically have a white, yellow, or pink color to the tissue. Histologically, a breast hamartoma may have an assortment of components previously described such as smooth muscle, adipose tissue, hyaline cartilage, or pseudoangiomatous hyperplasia, which are irregularly organized [8]. Despite its irregularity and disorganization, there is no malignant tissue interweaved within normal elements. However, in a study of 14 breast hamartomas in both males and females, one was found to have irregular borders and three showed myoid differentiation [10]. Myoid hamartomas belong to an uncommon subtype of breast hamartoma with low recurrence [11]. These hamartomas have smooth muscle cells that are histologically normal but irregularly and randomly distributed. While independent occurrence may not indicate a high risk for malignancy, the presence of both cellular differentiation and irregularity in the fibrous borders may require further histological studies.

Radiology remains a critical resource in differentiating malignant tumors from benign breast hamartomas. Imaging of breast hamartoma often shows a size-varying fatty mass with a thin radiopaque capsule, which makes it well circumscribed. The sonographic appearance is widely variable because of the varying amounts of fatty, fibrous, and glandular tissues. The most common radiologic characteristic is a well-circumscribed and compressible oval mass that is composed of predominantly hyperechoic fat [12]. The most common benign diagnosis of male breast mass is gynecomastia, which is typically diagnosed on mammogram as retroareolar fibroglandular breast tissue. Male breast cancer also presents with a painless subareolar mass. On imaging, breast cancer often presents as an irregular-shaped mass with ill-defined margins. Compared to a hamartoma, it does not have the “breast within a breast” characteristic. An important differential diagnosis of benign breast tumors includes lipoma which can present with similar radiologic features. Lipomas are benign, slow-growing tumors that come from fat cell overgrowth. On mammography, a fine capsule can allow for visualization of the radiolucent lesion. Sonographically, a lipoma may present as an echogenic mass with or without posterior acoustic enhancement. Fine linear striations parallel to the skin can also be seen in large lesions [13]. Compared with breast hamartomas, lipomas lack the mixed glandular and fibrous tissue and have a more predominantly adipose composition. The key defining feature between hamartomas from other malignant and benign growths of the breast is their well-circumscribed borders and “breast within a breast” tissue characteristic.

The exact pathogenesis of mammary hamartomas remains unclear, but there have been associated genetic mutations identified. A major genetic component of hamartomas includes Cowden disease, also known as multiple hamartoma syndrome. This condition is inherited in an autosomal dominant pattern and characterized by a mutation in phosphatase and tensin homolog gene (PTEN), a tumor suppressor gene. It is a subset of the PTEN hamartoma tumor syndrome [14]. Other symptoms of Cowden syndrome include mucocutaneous lesions, noncancerous brain tumors (Lhermitte-Duclos disease), intellectual disabilities, autism spectrum disorders, vascular abnormalities, and other benign diseases of the breast, thyroid, and endometrium. Hamartomas are benign as an isolated finding. However, their association with Cowden disease puts patients at a higher risk of developing breast, thyroid, and endometrial cancers. Therefore, a more thorough family history and physical examination may be warranted to investigate any possibility of association with Cowden syndrome. Surgical removal is an option for patients if the hamartoma leads to discomfort but is not mandatory in one with well-demarcated borders and halted growth. However, even with surgical excision, there are rare cases of hamartomas that result in recurrence [11].

## Conclusions

Breast hamartomas are most commonly detected in female patients but very rare in men. Since breast cancer can also occur in men, it is important to further investigate a tumor of undetermined significance. In this case of hamartoma in a male patient, the interval increase in tumor size was concerning for malignancy. Therefore, further imaging and ultrasound-guided core needle biopsy were performed for an accurate diagnosis. Although the exact etiology remains unknown, hamartomas may be seen in patients with genetic predispositions. Thus, clinical correlation with a patient’s past medical history may be helpful in the diagnostic process. The management of this benign condition includes reassurance and clinical follow-up without further interventions.
